# Acute Lymphoblastic Leukemia Cells Inhibit the Differentiation of Bone Mesenchymal Stem Cells into Osteoblasts In Vitro by Activating Notch Signaling

**DOI:** 10.1155/2015/162410

**Published:** 2015-08-03

**Authors:** Gui-Cun Yang, You-Hua Xu, Hong-Xia Chen, Xiao-Jing Wang

**Affiliations:** ^1^Key Laboratory of Developmental Diseases in Childhood, Chongqing 86-400014, China; ^2^Key Laboratory of Pediatrics in Chongqing, Children's Hospital of Chongqing Medical University, Chongqing 86-400014, China; ^3^Chongqing International Science and Technology Cooperation Center for Child Development and Disorders, Chongqing 86-400014, China

## Abstract

The disruption of normal hematopoiesis has been observed in leukemia, but the mechanism is unclear. Osteoblasts originate from bone mesenchymal stem cells (BMSCs) and can maintain normal hematopoiesis. To investigate how leukemic cells inhibit the osteogenic differentiation of BMSCs and the role of Notch signaling in this process, we cocultured BMSCs with acute lymphoblastic leukemia (ALL) cells in osteogenic induction medium. The expression levels of Notch1, Hes1, and the osteogenic markers Runx2, Osteopontin (OPN), and Osteocalcin (OCN) were assessed by real-time RT-PCR and western blotting on day 3. Alkaline phosphatase (ALP) activity was analyzed using an ALP kit, and mineralization deposits were detected by Alizarin red S staining on day 14. And then we treated BMSCs with Jagged1 and anti-Jagged1 neutralizing Ab. The expression of Notch1, Hes1, and the abovementioned osteogenic differentiation markers was measured. Inhibition of the expression of Runx2, OPN, and OCN and reduction of ALP activity and mineralization deposits were observed in BMSCs cocultured with ALL cells, while Notch signal inhibiting rescued these effects. All these results indicated that ALL cells could inhibit the osteogenic differentiation of BMSCs by activating Notch signaling, resulting in a decreased number of osteoblastic cells, which may impair normal hematopoiesis.

## 1. Introduction

Acute lymphoblastic leukemia (ALL) cells arise from the malignant proliferation of lymphoid precursors and occupy the bone marrow niche. Such niches, or bone marrow microenvironments, are known to regulate hematopoietic stem cell (HSC) survival, proliferation, and differentiation and thus play a crucial role in normal hematopoiesis. The malignant proliferation of leukemic cells disrupts normal bone marrow niches and creates abnormal microenvironments [1, 2], impairing normal hematopoiesis. In addition, these microenvironments are more favorable for leukemia stem cells because they support abnormal hematopoiesis [3] and mediate drug resistance [4]. However, the mechanisms underlying the leukemic cell-related disruption of bone marrow microenvironments are poorly understood.

Osteoblasts are an important part of the endosteal niche and play an essential role in the regulation of normal HSCs [5]. Osteoblastic cells can stimulate HSC expansion, maintain quiescence, and promote HSC mobilization. In addition, bone progenitor dysfunction can induce myelodysplasia [6], and even a single genetic change in osteoblasts can induce leukemogenesis [7]. These results demonstrate the important role that osteoblasts play in HSC regulation.

Bone mesenchymal stem cells (BMSCs) are recognized as bone marrow stroma stem cells and can differentiate into multiple cell lineages, including osteoblasts, adipocytes, and chondrocytes. The ultimate differentiation of BMSCs depends on signals from neighboring cells, and Notch signaling plays a critical role in cell differentiation during and after embryogenesis [8]. There are four Notch receptors (Notch1–4) and five known ligands (Jagged1 and 2 and Delta-Like1, 3, and 4), which are single-pass transmembrane proteins [9]. Notch-ligand interactions contribute to maintenance and renewal of adult tissues, such as the skin, the hematopoietic system, and the central nervous system. In the bone marrow, Notch signaling can maintain the stemness of BMSCs by suppressing osteoblast differentiation [10]. Meanwhile, the constitutive expression of the Notch1 intracellular domain impairs osteoblast differentiation and enhances adipogenesis in stromal cell cultures [11]. In addition, the activation of Notch signaling in osteoblasts causes osteopenia [12]. The abnormal activation of Notch pathways not only determines cell differentiation but also causes tumors. The oncogenic role of Notch signaling in T-cell malignancies has been well defined, and some B-cell malignancies express high level of Notch receptors and their ligands Jagged1 [13, 14]. However, whether abnormalities of Notch signaling in leukemia affect the differentiation of BMSCs to osteoblasts is unclear.

We hypothesized that leukemic cells can alter BMSCs differentiation via the activation of Notch signaling, resulting in a decreased number of osteoblastic cells, which may impair normal hematopoiesis. To test this hypothesis, we cocultured leukemia cells and BMSCs in vitro and observed the effect of leukemic cells on the osteogenic differentiation of BMSCs. Furthermore, we investigated whether Jagged1-induced Notch1 signaling played a key role in this process.

## 2. Materials and Methods

### 2.1. Patient Characteristics and Specimens

BM samples from 63 children with newly diagnosed ALL were recruited for this study. The diagnosis of ALL was based on morphology, cell immunophenotype, and cytogenetic analysis. The presence or absence of invasion osteoclasia or osteoporosis was determined by computerized tomography (CT) scans. The mRNA expression of Jagged1 was assessed by real-time RT-PCR. The study was approved by the institutional ethics committee of the Affiliated Children's Hospital of Chongqing Medical University in accordance with the Declaration of Helsinki, and written informed consent was obtained from patients and/or their legal guardians.

### 2.2. Cell Culture

BM samples from six children with newly diagnosed ALL and three healthy volunteers who donated bone marrow for transplantation were obtained for cell culture. The primary characteristics of these children in this study are presented in Table 1. Bone marrow mononuclear cells (BMNCs) were isolated by density gradient centrifugation (Lymphocyte separation medium, TBD, Tianjin, china) within 6 hours of sampling. Adherent cells were removed by plastic adherent culture and the remaining BMNCs were immediately used for the laboratory research. The BMNCs from healthy volunteers were cultured in DMEM/F12 (Gibco, USA) supplemented with 10% FBS (Gibco, Australia) in a 5% CO_2_-in-air incubator at 37°C. After 48 hours of adhesion, nonadherent cells were collected and stored in liquid nitrogen until use. The adherent cells were maintained in culture, with the medium being replaced every 2-3 days. Once the cultures reached 80–90% confluence, BMSCs were recovered by the addition of a 0.25% trypsin solution. All the experiments were performed with BMSCs harvested between the third and sixth passages.

### 2.3. Coculture

BMSCs were cultured alone or cocultured with ALL cells at a 1/10 ratio for 3 or 14 days to study the osteogenic differentiation of BMSCs. The cells were cultured in osteogenic induction medium, which consisted of growth medium supplemented with 0.1 mM dexamethasone (Sigma), 10 mM *β*-glycerophosphate (Sigma), and 50 mM vitamin C (Sigma). The expression of Notch1, Hes1, and the osteogenic markers Runx2, Osteocalcin (OCN), and Osteopontin (OPN) was assessed by real-time RT-PCR and western blotting on day 3. The ALP activity was analyzed using an ALP kit, and mineralization deposits were assessed by Alizarin red S staining on day 14.

### 2.4. Activation and Inhibition of Notch Signing in BMSCs

The recombinant rat Jagged1-Fc fusion chimera (R&D Systems) was dissolved in phosphate-buffered saline (PBS) at 10 *µ*g/mL and immobilized in flat-bottom 96-well plates overnight at 4°C, according to the manufacturer's protocol. Human IgG-Fc (R&D Systems) was used for the control. BMSCs were seeded in plates coated with Jagged1 or IgG-Fc at 10^4^ cells/well. After 2 days of culture, the medium was replaced with osteogenic induction medium, and the BMSCs were cultured for 3 or 14 days.

BMSCs were cocultured with ALL cells in osteogenic induction medium containing anti-Jagged1 neutralizing Ab (10 *µ*g/mL, GeneTex, USA) or vehicle (PBS) for 3 or 14 days. The expression of Notch1, Hes1, and the osteogenic markers Runx2, OCN, and OPN was assessed by real-time RT-PCR and western blotting on day 3. The ALP activity was analyzed using an ALP kit, and mineralization deposits were assessed by Alizarin red S staining on day 14.

### 2.5. Alkaline Phosphatase Activity and Alizarin Red Staining

ALP activity was detected using an ALP kit (Nanjing built Technology Co. Ltd, Nanjing, China) according to the manufacturer's protocol. Alizarin red S staining was used to visualize the mineralization deposits of BMSCs after different treatments on day 14. The ALL cells were removed, and the BMSCs were washed with cold PBS. They were then fixed in 10% formalin for 1 hour and stained with 2% Alizarin red S.

### 2.6. Real-Time Polymerase Chain Reaction (RT-PCR) Analysis

Total RNA was extracted using TRIzol reagent (Ambion, USA) and reverse-transcribed using the PrimeScript RT reagent Kit (TaKaRa, Japan). The mRNA expression of the genes encoding Jagged1, Notch1, Hes1, Runx2, ALP, OPN, OCN, and the housekeeping gene GAPDH was determined using the SYBR Green master mix (TaKaRa, Japan) on CFX 96 real-time PCR machine (BIO-RAD). The PCR conditions were as follows: 94°C for 30 s for the initial step; 39 cycles of 94°C for 5 s and the appropriate annealing temperature for 30 s; and extension in the last cycle for 5 s. The target expression was normalized to GAPDH and relative to a calibrator (control group). The relative expression was calculated using the formula 2^(−ΔΔCt)^. The primer sequences are listed in Table 2.

### 2.7. Western Blotting

BMSCs undergoing different treatments were washed with PBS and lysed in ice-cold lysis buffer with a protease inhibitor cocktail. Total protein and nuclear fractionation were performed using a whole protein or nuclear extraction kit (KENGEN Biotechnology, Nanjing, China). Equal amounts of protein (50 *µ*g) were fractionated by SDS-PAGE, transferred to polyvinylidene difluoride membrane (PVDF), and analyzed by immunoblotting using primary antibodies to Notch1 (Epitomics), Hes1 (Epitomics), Jagged1 (Abcam), Runx2 (Santa cruz Biotechology), OCN (Abcam), OPN (Epitomics), LaminB1 (Abcam), and *β*-actin. HRP-conjugated anti-rabbit or anti-mouse secondary antibodies were used as the secondary antibodies. The results were normalized to the loading control *β*-actin, and an ECL detection system was used for the data analysis.

### 2.8. Cytotoxicity and Apoptotic Assay

Cell viability of BMSCs with Jagged1 treatment was assessed by a colorimetric method (Cell Counting Kit-8; Beyotime, Beijing, China) using tetrazolium salt according to the manufacturer's procedure after long-term (1-2 weeks) treatment. The number of apoptotic BMSCs cells in short-term (3 days) treatment has also been evaluated by flow cytometry with AnnexinV-FITC Apoptosis Detection Kit (keygentec, Nanjing, china).

### 2.9. Statistical Analysis

Results are expressed as mean ± standard deviation. Statistical analysis was conducted using GraphPad Prism 6 software. Differences between groups were evaluated for statistical significance using a one-way analysis of variance; *P* values less than 0.05 were considered statistically significant. All experiments were repeated in triplicate.

## 3. Results

### 3.1. ALL Cells Inhibit the Osteogenic Differentiation of BMSCs

**Figure 1:**
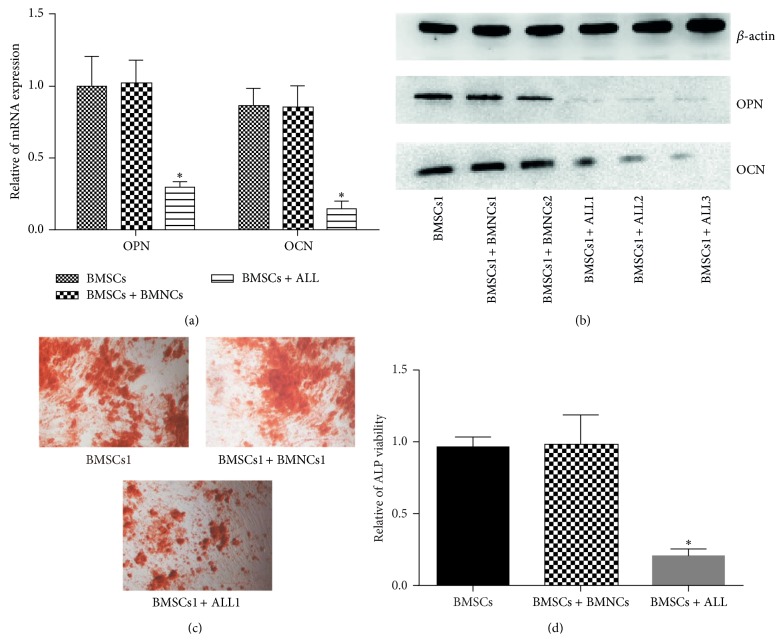
Effect of ALL cells on osteoblast differentiation of bone mesenchymal stem cells (BMSCs). BMSCs obtained from healthy bone marrow mononuclear cells (BMNCs) were cocultured with acute lymphoblastic leukemia (ALL) cells from six ALL patients or BMNCs from three healthy donor in osteogenesis induction medium for 3 days or 14 days. (a) The mRNA expression of Osteopontin (OPN) and Osteocalcin (OCN) were analyzed using real-time RT-PCR. (b) OPN and OCN protein were assessed by western blot analysis after 3-day coculture. (c) Calcium deposits were detected using von Kossa staining. (d) Alkaline phosphatase (ALP) levels were detected using an ALP kit (^*^ *P* < 0.05 versus BMSCs cultured alone or coculture with BMNCs).

The effect of ALL cells on the osteoblast differentiation markers was investigated. We used a coculture system with ALL cells and confluent BMSCs obtained from healthy volunteers. In these coculture systems, the osteogenic differentiation of BMSCs was assessed by ALP activity, the expression of OPN, and OCN and mineralization. Firstly, it was found that ALL cells, but not the normal BMNCs, reduced OPN and OCN mRNA expression in BMSCs after 3-day coculture (Figure 1(a)). OPN and OCN protein expressed by BMSCs were also consistently inhibited (Figure 1(b)). Furthermore, the ALP activity of BMSCs cocultured with ALL cells was significantly lower than that with normal BMNCs after 14 days (Figure 1(d)). Lastly, the mineralization in cocultured BMSCs after 14 days was assessed by Alizarin red S staining. Significant reduction of the mineralization levels was observed in BMSCs cocultured for 14 days with ALL cells but not in the control BMNCs (Figure 1(c)). Taken together, these results indicate that ALL cells significantly inhibit the osteogenic differentiation of BMSCs. No difference in the inhibitory effect of ALL cells was observed in the coculture systems.

### 3.2. ALL Cells Activate Notch Signaling in Cocultured BMSCs

**Figure 2:**
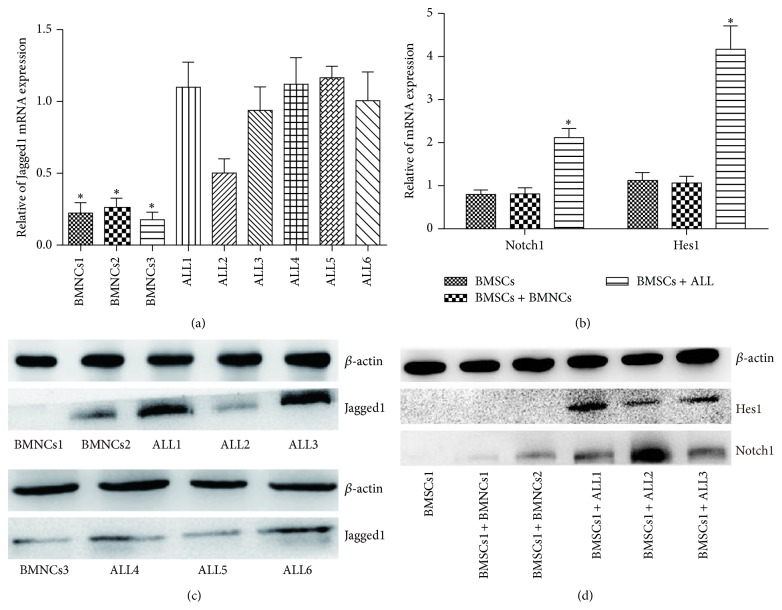
ALL cells activate Notch signaling in BMSCs in coculture. (a), (c) The Jagged1 expression levels in ALL cells and normal BMNCs were evaluated by real-time RT-PCR and western blotting (^*^ *P* < 0.05 versus BMNCs). (b), (d) The mRNA and protein expression levels of Notch1 and Hes1 in BMSCs were analyzed using real-time RT-PCR and western blot analysis after 3-day cocultured with ALL cells or BMNCs (^*^ *P* < 0.05 versus BMSCs cultured alone or coculture with BMNCs).

We further studied the expression and activation levels of Notch signaling in the process of the osteogenic differentiation of BMSCs under coculture conditions. First, the Jagged1 expression levels in ALL cells and normal BMNCs were evaluated by real-time RT-PCR and western blotting. Results showed that the expression of Jagged1 was significantly higher in ALL cells than in BMNCs (Figures 2(a) and 2(c)). Meanwhile, Notch1 expression in the BMSCs cocultured with ALL cells was significantly higher than that in the control BMNCs (Figures 2(b) and 2(d)), suggesting that Notch1 expression is negatively correlated with the osteogenic differentiation of BMSCs. Consistent with the observed Notch1 levels, the expression of Hes1, which is a target gene of Notch pathway, was also markedly increased in cocultured BMSCs. Thus, ALL cells can activate Notch signaling in BMSCs and suggest a negative correlation between Notch signaling and osteogenic differentiation of BMSCs.

### 3.3. Jagged1 Overexpressed in ALL Cells from Leukemia Children with Invasion Osteoclasia or Osteoporosis

To assess whether the invasion osteoclasia or osteoporosis is due to the Jagged1 overexpressed in ALL cells, the Jagged1 expression levels was evaluated in ALL cells from 63 leukemia children with or without invasion osteoclasia or osteoporosis by real-time RT-PCR. A significant overexpression of Jagged1 was observed in leukemia children with invasion osteoclasia or osteoporosis compared with those who did not have invasion osteoclasia or osteoporosis (Figure 6(c)).

### 3.4. Recombinant Notch Ligand Jagged1 Impaired the Osteogenic Differentiation of BMSCs

**Figure 3:**
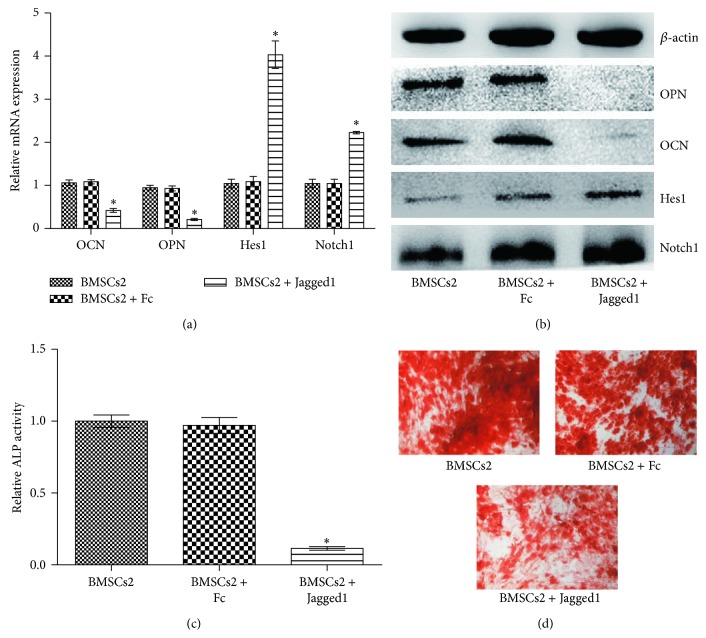
Effect of recombinant protein Jagged1 on osteoblast differentiation of BMSCs. We cultured BMSCs on immobilized soluble Jagged1 ligand in osteogenic induction medium for 3 days or 14 days, with Ig G-Fc as the control. (a-b) The mRNA and protein expression levels of osteogenic differentiation markers OPN and OCN, and Notch1 and Hes1 were analyzed using real-time RT-PCR and western blot analysis after 3-day (^*^ *P* < 0.05 versus BMSCs2 cultured alone). (c) ALP levels were detected using an ALP kit (^*^ *P* < 0.05 versus BMSCs2 cultured alone). (d) Calcium deposits were detected using von Kossa staining.

The cooccurrence of the enhanced Notch expression and impaired osteogenic differentiation by BMSCs cocultured with ALL cells prompts us to further investigate the role of Notch signaling in this process. We cultured BMSCs on immobilized soluble Jagged1 ligand in osteogenic induction medium for 3 days, with IgG-Fc as the control. To determine whether Jagged1 stimulates Notch activation, Notch1 and Hes1 expression were analyzed in BMSCs. The results showed that Notch1 and Hes1 levels were increased by Jagged1 treatment compared with the controls (Figures 3(a)-3(b)). Osteogenic differentiation markers were also assessed, as previously mentioned. The ALP activity was significantly lower in the Jagged1-treated cells (Figure 3(c)). In addition, the mRNA and protein expression levels of OPN and OCN were reduced in the Jagged1 group (Figures 3(a)-3(b)). Consistent with these findings, Jagged1 protein inhibited osteogenic mineralization (Figure 3(d)). These results imply that Notch signaling is critical for the impairment of the osteogenic differentiation of BMSCs.

### 3.5. Anti-Jagged1 Neutralizing Ab Rescued the Osteogenic Differentiation of Cocultured BMSCs

**Figure 4:**
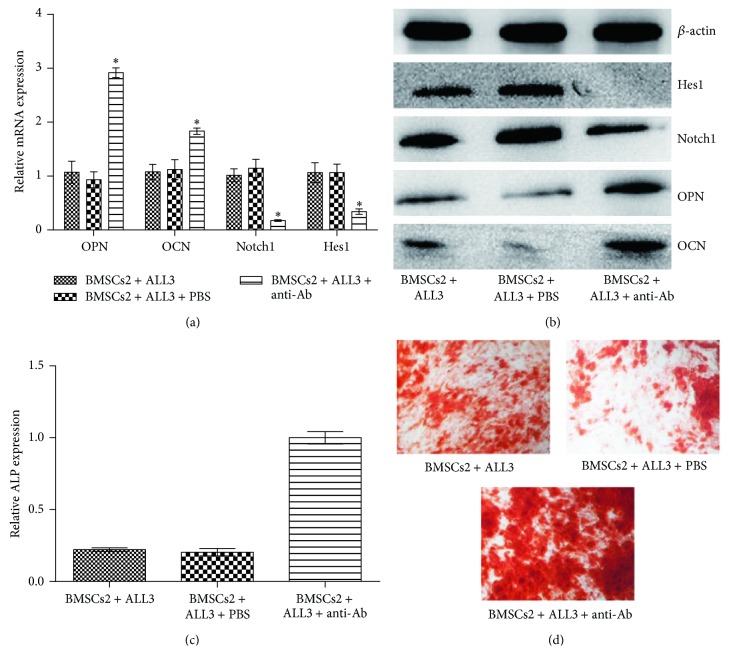
Effect of anti-Jagged1 neutralizing Ab on osteoblast differentiation of BMSCs in cocultures. BMSCs2 and ALL cells were cocultured with or without anti-Jagged1 neutralizing Ab under osteogenic conditions for 3 days or 14 days. (a-b) The mRNA and protein expression levels of osteogenic differentiation markers OPN and OCN, and Notch1 and Hes1 were analyzed using real-time RT-PCR and western blot analysis after 3-day coculture. (c) ALP levels were detected using an ALP kit (^*^ *P* < 0.05 versus BMSCs2 cocultured with ALL cells). (d) Calcium deposits were detected using von Kossa staining.

To further confirm the role of Notch signaling in this process, anti-Jagged1 neutralizing Ab was introduced into coculture systems with BMSCs and ALL cells to inhibit Notch signaling. As shown in Figures 4(a)-4(b), Notch1 and Hes1 expressions were clearly inhibited by anti-Jagged1 neutralizing Ab. After BMSCs and ALL cells were cocultured with anti-Jagged1 neutralizing Ab under osteogenic conditions for 3 days, the mRNA and protein expression levels of OPN and OCN were elevated in the anti-Jagged1 neutralizing Ab group (Figures 4(a)-4(b)). In addition, the ALP activity was significantly higher than that without anti-Jagged1 neutralizing Ab (Figure 4(c)). Consistent with these findings, the inhibitory effect on Notch signaling promoted osteogenic mineralization. These results suggest that inhibition of Notch signaling can rescue the impaired osteogenic differentiation of BMSCs.

**Figure 5:**
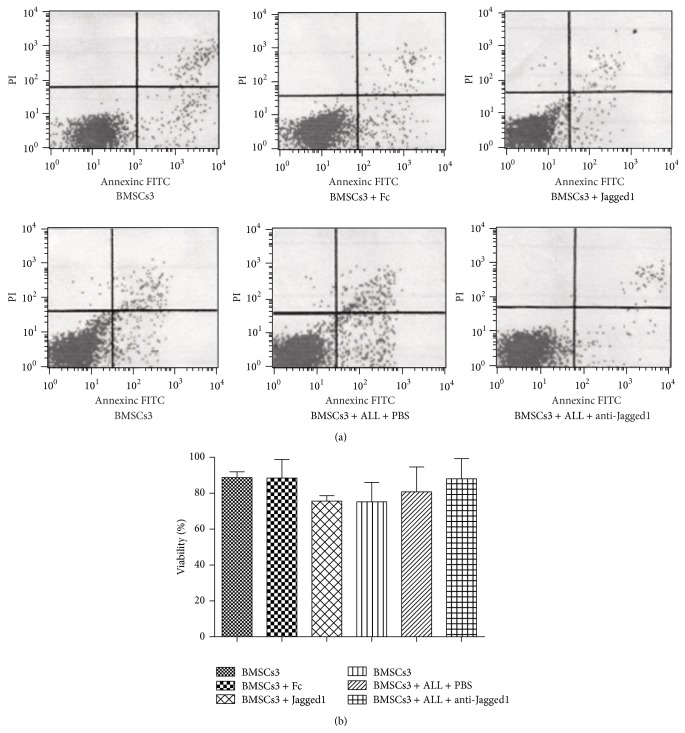
No toxic and apoptotic effect on BMSCs in the Jagged1 treatment. (a) The presence of both death and apoptotic BMSCs has been investigated by flow cytometry after 3 days in the presence and absence of recombinant Notch ligand Jagged1 or anti-Jagged1 neutralizing Ab. (b) The viability of BMSCs in the Jagged1 treatment using a cytotoxic assay after 14 days.

To exclude that the inhibitory effect observed on BMSCs in our Jagged1 treatment could be due to toxicity, we tested the viability of BMSCs by flow cytometry and confirmed that no toxic or apoptotic effect was present in BMSCs after 3 days (Figure 5(a)). And then we have evaluated the viability of BMSCs in the presence and absence of recombinant Notch ligand Jagged1 or anti-Jagged1 neutralizing Ab using a cytotoxic assay. The viability of BMSCs was evaluated after 7 and 14 days. No significant reduction of BMSCs viability was observed at any time point.  Figure 5(b) shows the percent of cell viability at 2 weeks.

### 3.6. Effect of ALL Cells on Runx2 Expression in BMSCs

**Figure 6:**
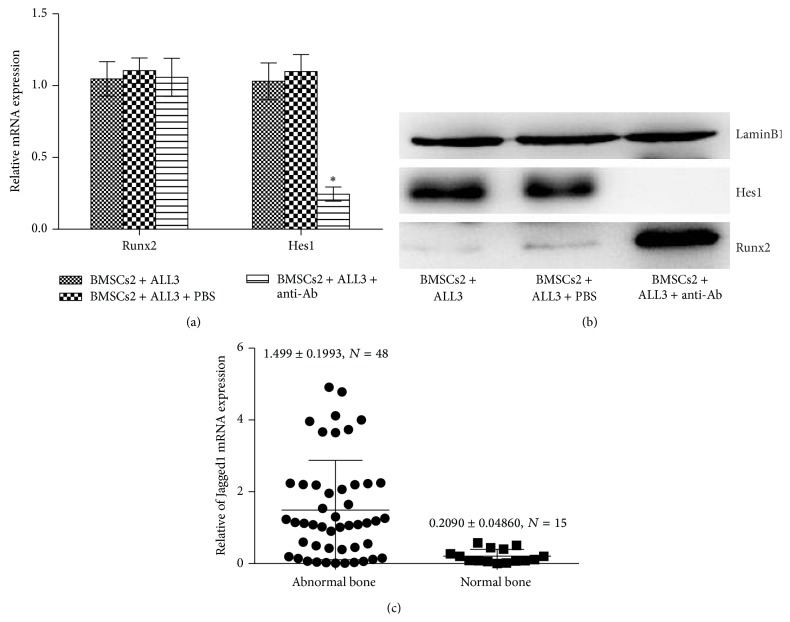
(a) The mRNA expression of Runx2 and Hes1 was evaluated by real-time RT-PCR in BMSCs2 after 3-day coculture with ALL cells with or without anti-Jagged1 neutralizing Ab (^*^ *P* < 0.05 versus BMSCs2 cocultured with ALL cells). (b) The protein expression of Hes1 and Runx2 were assessed. (c) The Jagged1 expression levels were evaluated in ALL cells from 63 leukemia children with or without invasion osteoclasia or osteoporosis by real-time RT-PCR. Abnormal bone represented leukemia children with invasion osteoclasia or osteoporosis.

To investigate whether ALL cells could affect the expression of the critical osteoblast transcription factor Runx2, BMSCs with ALL cells were cocultured. First, we found that Runx2 mRNA expressed by BMSCs was not modified after 3 days of coculture (Figure 6(a)). However, Runx2 protein expression in BMSCs nucleus, as evaluated by nuclear extract western blots, was modified in the presence of ALL cells (Figure 6(b)). To further investigate the connection between Notch signaling and Runx2, anti-Jagged1 neutralizing Ab was applied to inhibit Notch signaling and results showed that Runx2 mRNA expressed by BMSCs was not modified but protein level was elevated (Figures 6(a)-6(b)). In summary, Notch signaling showed an inhibitory effect on Runx2 protein level but not on Runx2 mRNA expression.

## 4. Discussion

The inhibition of normal hematopoiesis is partially responsible for the impairment of the bone marrow microenvironment in leukemia [15, 16]. Osteoblasts have long been known as important parts of the bone marrow microenvironment and have been known to support HSCs in vitro. Recent data suggest that BMSCs give rise to cells of the osteogenic lineage, and studies indicate that leukemic cells can inhibit osteoblastic cell function and decrease osteoblastic cell numbers [17]. However, how ALL cells implement this process is poorly understood.

Our data demonstrate that the osteogenic differentiation of BMSCs is inhibited by ALL cells, as demonstrated by the decreased expression of osteogenic markers. The inhibitory effect of ALL cells on the osteogenic differentiation of BMSCs might explain the decreasing number of osteoblasts, which leads to the destruction of the bone marrow microenvironment and impairs support of normal hematopoiesis. This conclusion is in agreement with an in vivo study showing that osteoprogenitor numbers are decreased in the long bones of leukemic mice [17, 18] and increased osteoblasts in mouse models of acute leukemia decrease leukemia blasts in the bone marrow and reestablish normal hematopoiesis [18]. Meanwhile, numerous studies have shown a decrease in the markers of bone formation in pediatric acute leukemia cases at diagnosis before corticosteroid treatment [19]. The inhibition of ALL cells in the osteogenic differentiation of BMSCs is further supported by previous studies that maintaining a pool of mesenchymal progenitors led to a deficit in osteoblast production and resulted in precipitous bone loss [10].

Some previously published data have shown that leukemia BMSCs exhibit similar differentiation potential compared with BMSCs from health donors [20]. These contradictory results could be explained by the possibility that the effect of ALL cells on BMSCs is reversible. Once leukemia cells are removed, the BMSCs return to normal, as shown by the increased expression of bone formation markers after the reduction in disease burden by chemotherapy [21]. In addition, a recent study demonstrated that osteoblasts regulated ALL cells dormancy and protected them from cytotoxic chemotherapy [22]. One potential explanation for these dissimilar results is that leukemic cells not only inhibit osteoblastic cell function and decrease osteoblastic cell numbers, but also can change the osteoblast, which further create a favorable niche for ALL cells.

The mechanism by how ALL cells inhibit osteogenic differentiation of BMSCs was also investigated in this study. We focused on Notch signaling since this pathway regulates osteogenic differentiation. Previous studies have demonstrated that Notch signaling maintains a pool of mesenchymal progenitors by suppressing osteoblast differentiation [10]. The osteogenic differentiation potential of mesenchymal stem cells can be promoted by inhibiting Notch1 activity in vitro [23]. In addition, abnormal Notch signaling is associated with cancer, including leukemia. Studies have indicated that Notch1 and Jagged1 are highly expressed in B- and T-cell-derived Hodgkin's lymphoma and anaplastic large cell tumor cells [14]. Notch ligands Jagged1/2 and Delta ligands are expressed in BMSCs and, in the context of leukemia, BMSCs can enhance Notch signaling in human B-ALL cells via Jagged1 and rescue B-ALL cells from drug-induced apoptosis in vitro [24]. However, whether the abnormal Jagged1 in ALL cells affects the osteogenic differentiation of BMSCs is unknown.

In accordance with previous reports [13, 14], we observed that Jagged1 was highly expressed in ALL cells compared with normal BMNCs. Consistent with Notch-ligand interactions, enhanced signaling was observed in BMSCs after coculturing with ALL cells, as demonstrated by the increased expression of Notch1 and Hes1. In addition, we found that the expression of osteogenic markers was decreased and, once anti-Jagged1 neutralizing Ab was added to the coculture system, the osteogenesis potential of BMSCs was regained, suggesting that ALL cells can inhibit the osteogenic differentiation of BMSCs by activating Notch signaling. The involvement of Notch signaling in the osteogenic differentiation of BMSCs is further supported by the evidence that Notch signaling stimulation by a soluble Jagged1 ligand decreases the expression of osteogenic markers. Moreover, the mRNA expression of Jagged1 in ALL cells supports our in vitro study. Children with invasion osteoclasia or osteoporosis highly expressed Jagged1 in comparison with children without invasion osteoclasia or osteoporosis. This evidence suggests that the overexpressed Jagged1 in ALL cells might activate the Notch signaling in BMSCs and lead to a reduction of the number of osteoblastic cells.

Runx2 is a crucial transcription factor in osteogenic differentiation, regulating the expression of osteoblast markers such as ALP, OCN, and OPN [25]. Hes1, which is downstream of Notch signaling, may mediate the Notch-induced inhibition of osteoblast differentiation by inhibiting Runx2 activity [26]. Similarly, in our coculture system, we found that ALL cells decreased Runx2 protein expression. This finding is in agreement with a previous study showing that the expression of Runx2 in Notch1 knockdown BMSCs was upregulated. In contrast, human myeloma cells only block Runx2 activity, without modifying Runx2 expression in coculture system with a mesenchymal/stromal cell line [27]. This result could be explained by the different experimental system applied by different researchers and the potential involvement of other signaling pathways.

In conclusion, our findings indicate that abnormal Notch signaling not only induces leukemia cell proliferation but also inhibits the osteogenic differentiation of BMSCs, which further disturbs normal hematopoiesis. The prevention of abnormal Notch signaling in BMSCs would be beneficial for the restoration of normal hematopoiesis. Furthermore, an in vivo study on the mechanisms involved in BMSCs differentiation into osteoblast and other lineages in the context of ALL is urgently required.

## Figures and Tables

**Table 1:** The primary characteristics of ALL children at diagnosis.

Sample number	Sex	Age (months)	% of BM blast	Immunophenotype	Cytogenetics
1	F	44	98	common B-ALL	46XX
2	F	75	98.5	common B-ALL	46XX
3	M	133	97.5	T-ALL	46XY
4	M	82	86.5	common B-ALL	46XY
5	F	37	99	Pre-B	46XX
6	M	13	94.5	Pre-B	46XY

M, male; F, female.

**Table 2:** Primers sequences for real-time RT-PCR analysis.

Gene	Length	Annealing temperature (°C)	Sequence
OCN	81	60.7	GGTGCAGCCTTTGTGTCCA
			GGCTCCCAGCCATTGATACA

OPN	81	63	GGCCGAGGTGATAGTGTGGTT
			AGCATCAGGGTACTGGATGTCA

Hes1	313	63.5	AAAATGCCAGCTGATATAATGGAG
			GGTCTGTGCTCAGCGCAGCCGTCA

Notch1	76	63.5	CGGGTCCACCAGTTTGAATG
			GTTGTATTGGTTCGGCACCAT

Runx2	101	62.3	TTATTCTGCTGAGCTCCGGAA
			AACTCTTGCCTCGTCCACTCC

Jagged1	164	60	GCTGCCTTTCAGTTTCGC
			CGCCCGTGTTCTGCTTCA

GADPH	114	54	CCACATCGCTCAGACACCAT
			GGCAACAATATCCACTTTACCAGA

## Abbreviations

BMSCs: Bone mesenchymal stem cells
ALL: Acute lymphoblastic leukemia
OPN: Osteopontin
OCN: Osteocalcin
ALP: Alkaline phosphatase
HSC: Hematopoietic stem cell
CT: Computerized tomography
BMNCs: Bone marrow mononuclear cells
PBS: Phosphate-buffered saline
PVDF: Polyvinylidene difluoride membrane
RT-PCR: Reverse transcription-polymerase chain reaction.
